# Alkalies

**Published:** 1873-02

**Authors:** H. L. Sage


### Dental Register.

Vol. XXVII.] FEBRUARY, 1873. No. 2.]

### COMMUNICATIONS.

### Alkalies.

BY H. L. SAGE, D. D. S.

In regard to the relative effects of acids and alkalies upon the teeth, doubtless every one will admit, that the former are much more destructive in the aggregate than the latter, for this reason, if no other, that acids are more generally present in the mixed saliva of the mouth than alkalies. That alkalies *never* attack the structure of the teeth, is not probable—and it may be, that in those comparatively rare cases where caries is produced by the action of alkalies, the destruction is more complete and thorough, than when an acid is the agent.

Whether the action is vital or chemico-vital, is a question. It is, probably, in some cases, both, while in others, it may be either vital or chemical. In Garretson's Oral Diseases and Surgery, page 164, we read: "In these depressions, (referring to the teeth) lodge and decompose the various ingesta of diet, acting of course as irritants, and producing, as I believe, just the same character and effect as would be produced in any bone; that is inflammation, yet modified of course by differences, which exist between the tooth bone and ordinary bone; it is, however, inflammation in the one case and inflammation in the other; the matter of *acids* and *alkalies* has little to do with the matter, excepting as they are agents of irritation," thus admitting, that alkalies may have an influence in the production of caries, by causing inflammation in the dentine. The action then becomes vital, by lessening the ability of the teeth to withstand the destructive effects of chemical agents.

He only casually alludes to the agency of alkalies in this direction, and this is almost entirely overlooked by most, if not all, dental writers.

Tomes also considers caries a chemico-vital action. He alludes, on page 372 of *Dental Surgery*, to "those agents which may be regarded as capable of acting in the double capacity of depriving the dentine of its normal powers of resistance, and of producing its immediate decomposition"—and "that the vitality of dentine must have been lost before the tissues undergo decomposition." The consolidation of dentinal fibrils on the surfaces of carious cavities, he regards as "an attempt on the part of nature to circumscribe and limit the mischief."

Harris, on the other hand, scouts the idea, that caries of the teeth is influenced by inflammation of the dentine, or that the osseous structure of a tooth, (considered with regard to the fibrils) is capable of being inflamed, and regards it as the result of the action of chemical agents alone.

Prof. Taft sustains the chemico-vital theory.

The cause of the decomposition of dentine, is, he maintains, "either a lack of *vital power* to maintain the integrity of the organic structure, or the action of some agent having an affinity for a certain portion of the dentine more potent than that vital power." Impaired vitality, not only of the teeth, but of the general system, he regards as an indirect cause of caries. He also alludes to inflammation of the organic portion of the dentine, as being produced not only by local irritants, but as accompanying febrile, inflammatory, and other pathological conditions of the general system.

Regarding the action of alkalies, he states that they "will act upon the animal portion of the dentine and remove it; and in caries thus produced, the residue is friable and chalk-like"—and also, that acids, some alkalies, salts and sweets will cause irritation of the nerve-tissue.

Garretson also says: (Oral Diseases and Surgery, 1st edition, page 198) "Certain irritative conditions of the oral fluids should receive attention; thus, whether the fluids be too acid or too alkaline, it is alike injurious and should receive attention."

That alkalies may have some agency in the production of caries, not only by their local action upon the vital or organic portion of the dentine, but indirectly by their systemic influence, as in some general pathological conditions, is, until disproved, entitled to some consideration. In regard to the local action of alkalies, it would seem to be more potent in the teeth of young subjects, in which the organic matter is in larger proportion than in adult life, for, as age advances, the teeth may become more dense by the gradual calcification of the organic portion of the dentine, and in a measure the obliteration or filling up of the tubuli by a deposition of lime salts, leaving the calcareous portion in greater excess, than is the case in the more highly organized dental structures of the young.

Acids, it is claimed by some, will not act upon living tissue, or upon the mineral portion of healthy bone, when applied for the purpose of dissolving carious or necrosed bone. While it is true, that acids will seize upon the lime of a living tooth, which has a lower degree of vitality than bone, it is likewise true, that if the vital or organic portion be removed or destroyed by inflammation and death, whether effected by the action of alkalies or by other means, the remaining inorganic portion will be acted upon more readily by the secretions of the mouth, should they subsequently become acid, owing to this loss of vitality.

So too, if the fluids of the mouth are abnormally or excessively alkaline, and inflammation exists in the animal portion of the tooth, will it not be less capable of resisting the action of these alkaline fluids, than if the organic constituents were in a normal condition?

Suppose caries, even though superficial, were caused in the first instance by acid secretions, should the latter become changed to an abnormally alkaline condition, the teeth would probably continue to decay from the latter cause, owing to the organic portion having become exposed to the action of alkalies—though sound teeth bathed in *healthy* alkaline saliva will remain unaffected.

If bone be placed in a caustic alkali, the organic matter will be dissolved, leaving the lime. If hydrochloric acid be used, the lime will be dissolved leaving the gelatine. If a tooth be placed in an acid as weak as that contained in the acid or excessively acid, or alkaline or excessively alkaline secretions of the mouth, no visible effect will be produced on the enamel, for an indefinitely long period, in the one case or the other, and yet, if acids be present in the mouth, they will, in this weak state, decompose the lime of the tooth, or on the other hand, if the secretions of the mouth be excessively alkaline, they will act by dissolving the gelatine and fat. To be less positive, if the weak acid secretions of the mouth act upon the mineral portion of the tooth, why may not weak alkalies dissolve the animal portion, though if, out of the mouth, a tooth be placed in a weak alkaline solution, no effect will be produced?

Perhaps acid secretions will act more *readily* on the inorganic portion, than those which are excessively alkaline will, on the organic, the progress being, in the latter case, more tardy.

Thus, the excessive use of alkaline mouth washes, may prove deleterious by dissolving out the fat, and also the gelatine, from the enamel and dentine, leaving the surface soft and chalky.

A prominent dentist affirms that he has seen this effect produced by the use of sozodont.

Headland says, (Action of Medicines, page 145) "Alkalies dissolve fats," Page 146, "Alkalies likewise dissolve organic compounds of the albuminous group, and prevent the coagulation of fibrine."

Tomes says, (Dental Surgery, page 373) "Litmus paper applied in the cavity of a carious tooth, almost invariably gives strongly marked acid reaction, and thus furnishes evidence of the existence of an agent capable, if unresisted by the vitality of the dentine, of depriving that tissue of its earthy constituents, leaving the gelatine to undergo decomposition, favored by the heat and moisture of the mouth."

The ravages of caries were most extensive, in the case of a young man, in which the secretions of the mouth presented an *alkaline* reaction.

In this case, litmus paper, previously reddened by an acid, and applied in the *cavities* on the labial surfaces of the front (superior) teeth, showed an *alkaline* reaction. Cavities were present in almost every conceivable location, and, on the labial surfaces of the front teeth, were nearly cresentic in shape, besides forming, as expressed by Mr. Tomes, "An annular belt of softened tissue nearly around the teeth."

Those portions of the lingual surfaces, next to the gums, of the first and second inferior molars, did not escape the destructive effect of the agent or agents at work in this mouth—the cavities presenting horizontal, narrow and somewhat irregular strips of decomposed tissue, at or just above the junction of the enamel and cementum, which may be cited as a very rare case, of caries in this location. As in this case, the carious tissue was not as sensitive as would be expected by the appearance, may not this be attributed to the absence of acid secretions in the cavities?

The sensitiveness resides in the organic portion of the dentine. Alkalies act upon this by dissolving it, which may account for the absence of the usual amount of sensitiveness in the above case.

Again, may not the presence of ammonia in the saliva or in the breath, produce caries in rare cases, by its action upon the vital portion of the tooth? In the case before referred to, if litmus paper, reddened by an acid, was held over the gums of the front teeth, a vapor would be immediately formed indicating the presence of a volatile alkali. Was the latter, if such it was, present in the breath or in the saliva? Most probably in the former.

If in either the breath or the saliva, the inference would be, that ammonia was present in the blood, or produced by the decomposition of organic matter in the stomach.

Piggott, while admitting that ammoniacal saliva exists, states that it is rare; that "it is dingy and clouded, with a very strong alkaline reaction," and that "neuralgia and nervous disturbances generally have a remarkable connection with alkalinity of the saliva" (Dental Chemistry and Metallurgy page 214 and 215.)

Whether or not ammonia exists in the blood, is a matter of controversy. In Headland's Action of Medicines, page 389, the author says: "According to Dr. B. W. Richardson, the existence of free ammonia in small quantity in healthy blood is the chief or only cause of its alkaline reaction." etc.

Page 388, "Ammonia is capable of acting chemically as an alkali in the system." Page 391, "Experimenters differ on the question of the presence of ammonia in the breath and perspiration. The former is more readily examined. Richardson, with Viale and Latini, states that ammonia is present in the healthy breath. Reuling, on the contrary, finds etc. But this ammonia in the breath, an index of that in the blood, is increased, he says, in low fevers and albuminuria." Page 391, "Ammonia is volatile and soluble in air; and tends for that reason to pass off freely from those secreting surfaces which are immediately in contact with the atmosphere."

If this be true, may it not pass off through the mucous follicles of the oral cavity as well as in the breath, and if in excess, exert some influence in the production of caries? Page 143, "The natural alkalinity of the blood is due to the presence of free soda and ammonia (see page 135.) Potash, when given as a medicine, must liberate these weaker alkalies from their saline combinations. Thus potash rarely, if ever, exists free (or carbonated) in the blood. It acts by setting free soda or ammonia." Question: will not potash, when taken in the food, liberate ammonia, and thus, if in excess, produce injurious effects upon the teeth?

Alkalies, in excess, are said to retard nutrition and cause spareness of the system. Thus it would seem, that the influence would extend to the osseous system and the teeth, and thus cause deterioration in this direction, by checking the nutrient action of materials, which would otherwise go to maintain the integrity of the latter. In some diseases, in which alkalies are in excess in the blood, this might be one of the effects—as in some kinds of fever.

Headland (page 136) thus refers to this excess in fevers: "In the yellow fever of tropical climates, an excess of alkali in the blood was discovered some years since by Dr. Blair. This alkali seems to be ammonia."

From the fact that some of the English physicians treated typhus and typhoid fevers, with the greatest ratio of success, with mineral acids, it would seem to confirm the truth of the above theory. "Of 230 cases of continued fever in St. Mary's Hospital, Dr. Chambers treated 109 on "general principles," 121 with hydrochloric acid. Of the 109, 23 died; of the 121, only four died. Continuous liquid nutriment of beef tea and milk, was given along with the acid. Dr. Henderson found at Shanghai that the mortality from continued fever was reduced by the employment of the acid from 28 to 7 per cent.

Mr. Day, of Stafford, has found nitric acid of great use in malignant scarlatina."

(See also page 138 on the use of vegetable acids) "In fevers of a low type, when there is a tendency to alkalinity, the vegetable acids may act directly by neutralizing the alkali in the blood," etc.

It is not unreasonable to suppose, that this excessive alkalinity of the blood, during fevers and some other diseases, may cause deterioration of the teeth, aside from the action of medicines or the loss of nutrition, for it might, as before stated, be eliminated not only by the salivary glands, but, if a volatile alkali, through or upon the mucous surfaces, and by the breath, and thus act injuriously in a variety of ways.

It appears that the salivary glands are very sensitive to physiological and pathological conditions, or changes of the system. According to Lehmann, many mineral and organic substances are rapidly eliminated by them, far more rapidly than by the urine, and before they could be separated by the kidneys from the mass of the blood. It would thus appear, from the foregoing statement, that an excess of acid or alkali in the system, would be likely to be at once indicated by the saliva, and many alkaline constituents of food would thus be expelled by these glands.

Lehmann also states, that alkalinity of the saliva is often associated with cholera and Bright's disease, to which is given the name Uræmia. He has always found the vomited contents of the stomach strongly alkaline, and rich in carbonate of ammonia. (See Lehmann's Physiological Chemistry, vol. I. page 424, 426 and 448.)

"According to Wright," says Lehmann, the quantity of alkali in the saliva is increased after the use of fatty, aromatic, acid, spirituous, and more particularly, indigestible kinds of food and drink."

The acids generated in the stomach in certain kinds of dyspepsia, and thrown off therefrom by eructation or otherwise, will render the saliva acid, as well as act upon, or corrode and decompose the enamel, etc., while that kind of indigestion associated with excessive alkalinity of the contents of the stomach, will also inflnence the character of the secretions of the mouth, and might thus render them abnormally alkaline, with the accompanying deterious action upon the teeth.

Intermittent fever is said to be a blood disease, caused by exposure of the system to a peculiar poison generated in miasmatic districts, by decaying vegetable matter. Now, one of the products of the decomposition of the latter is ammonia. Perhaps the inhalation of this in large quantities, and its absorption by the blood, (aside from or together with the poisonous gases thrown off by decaying organic matter, and inhaled) may have some effect in producing this disease.

Headland refers to the "increasing excretion of ammonia by the breath and skin in low fevers and eruptive disorders."

Whether or not an excess of alkalies is the cause, or only an accompanying condition, or an effect of many diseases, it is evident that their action is two-fold. It may be, by impairing the vitality of the system, and thus be a predisposing cause of caries. It may be that, either by elimination in excess by the salivary glands, the mucous follicles of the oral cavity, their exhalation by the breath, and their presence in undue quantity in the fluids of the mouth, they act directly upon the vital structures of the teeth. Then again, their excessive local or constitutional employment may be productive of caries.

Whether or not, as before intimated, an excessive alkalinity of the general system may be an *effect* or a *cause* of many diseases thereof, may not be apparent. That it may be, in some instances, a predisposing, and in others, a direct cause or excitant of diseases in the dental organs, is more capable of proof. That in isolated cases it may act simultaneously in both directions, would seem very probable, from the possibility of its acting in either. But it is presumed, that an excessive alkalinity of the saliva, of the blood, or of the organic matters of the stomach, is not always confined to persons whose systems are affected by any of the diseases before enumerated. It may be, as the case of the young man referred to in this paper would go to show, that an individual enjoying a good degree of health, may experience the most thorough and destructive type of caries in his dental organs, by the direct or indirect action of alkalies.

Much has been written concerning the influence or action of tobacco upon the teeth, more especially in connection with chewing—most maintaining that it is inert—others, that it renders the gums unhealthy, softens the dentine, etc. But there is another aspect of the question which has been overlooked. The vapor of burning tobacco is said to contain, in considerably quantity, carbonate of ammonia, which is, according to Headland, largely absorbed by the mucous surfaces of the mouths and lungs of smokers. This, he says, must be viewed as a course of alkaline medication, with risk or advantage attending it, according as the humors are alkaline or the reverse—it being in the former case injurious, or in the latter beneficial.

Thus will not the excessive smoking of tobacco, by this absorption of the alkaline constituents by the mucus membrane, vitiate and change the character of the secretions of the mouth to such an extent, as to act injuriously upon the structure of the teeth?

The breath and saliva of habitual smokers is also ammoniacal, at least the former; and thus, large quantities of ammonia are evolved and thrown upon, or come in contact with the teeth. The question is of importance—is smoking (viewed in this light) injurious?

Next, comes a consideration of the action of tobacco (used in this form) as a *predisposing* cause of caries.

Persons overburdened with fat, sometimes resort to smoking as a remedy, the effect being to reduce the excess. This course of "alkaline medication," if it has the effect to dissolve fat, retard nutrition and cause spareness of the system, might, in the case of individuals in whom a tendency to spareness exists, by producing these effects, be productive of injury, by causing a withholding of certain elements of nutrition from the teeth, and thus render them less capable of withstanding the effects of chemical agents.

Of the effect of acids upon the dental structures, every one is cognizant—and it is hardly a matter of controversy, excepting as to whether in caries thus produced it is simply chemical, or a two-fold chemico-vital action—the weight of opinion and of proof being in favor of the latter theory.

If, as Harris takes the ground, the action of caries is merely chemical, or according to the theory of Tomes, Tait, and Garretson, chemico-vital, alkalies, as before intimated, would, in either case, seem to play an important part in causing caries of the teeth, whether by acting directly upon the organic portion, by causing irritation, or by retarding nutrition, and thus lessening the powers of resistance to the action of chemical agents. So little is known or said about the direct or indirect action of alkalies upon the teeth, or whether they have such action, that much of what is here gathered, is a mere *resume* of what may have a bearing upon the subject. It is believed by most, that alkalies have no agency in the production of caries, which doubtless accounts for the absence or dearth of literature upon the subject, together with the fact, it may be, that but little is known in regard to it, and it may seem presumptuous, to tread upon ground touched so lightly by minds capable of making close and thorough investigation. But many things not positively known are assumed—and assumptions, inquiries, and investiagtions often open the way for the clearing up of mysteries, and the development of facts.

Our profession, as a *specialty*, is yet in its infancy; and it is not surprising, that much obscurity should yet envelop many theoretical questions, and that the effect of many pathological conditions of the general system, upon the development of the teeth and maintenance of their integrity, should not be fully comprehended. The medical profession even, with which is allied the dental, though of great antiquity, is not yet full fledged. But the continued evolution of light, will most assuredly develop more intelligent methods of practice, based upon a more correct knowledge of the causes, effects, influences and workings of abnormal conditions, and the attendant direct and indirect agencies, with the specific action of each upon certain tissues, and the elements entering therein.

